# Management of genitourinary syndrome of menopause using diode vaginal laser combined with pelvic floor chair

**DOI:** 10.6026/973206300211606

**Published:** 2025-06-30

**Authors:** Minie Anand, Winie Gautam, Manjusha Mohan, Nikhil Anand, Sinha Manjari

**Affiliations:** 1 Department of Obstetrics and Gynecology, Madhubani Medical College & Hospital, Keshopur, Bihar 847212, India; 2 Department of Pathology (Consultant Pathologist), Ama Diagnostic Centre Pvt Ltd., Lucknow, Uttar Pradesh, India; 3 Department of Conservative Dentistry, Chandra Hospital and Research Centre, Safedabad, Barabanki, Uttar Pradesh, 225001, India; 4 Department of General Medicine, Jawaharlal Institute of Postgraduate Medical Education and Research, Jipmer Campus Rd, Jipmer Campus, Puducherry, 605006, India

**Keywords:** Genitourinary syndrome of menopause, diode vaginal laser, pelvic floor chair, non-hormonal therapy, vaginal atrophy, urinary incontinence, sexual dysfunction, female sexual function index (FSFI), visual analogue scale (VAS), questionnaire urinary incontinence short form (ICIQ-UI SF)

## Abstract

Genitourinary syndrome of menopause (GSM) is a common condition in postmenopausal women, marked by vaginal atrophy, dyspareunia, urinary incontinence and reduced sexual function. This study evaluated 50 women aged 45-70 years receiving 1470 nm diode laser therapy monthly for three months and pelvic floor chair therapy twice weekly for six weeks. Significant improvements were observed, with VAS scores for vaginal dryness and dyspareunia decreasing markedly, FSFI scores improving and urinary incontinence symptoms reducing as reflected by lower ICIQ-UI SF scores (all p < 0.001). The combined treatment demonstrated substantial symptomatic relief and enhancement of quality of life. Thus, a safe and effective non-hormonal approach for managing GSM is suggested.

## Background:

Genitourinary syndrome of menopause (GSM) is a common condition in postmenopausal women, defined by a variety of symptoms including vaginal dryness, burning, itching, dyspareunia, urinary urgency, frequency and incontinence, all of which have a notable effect on quality of life [1]. These symptoms are mainly caused by decreased estrogen levels and loss of pelvic floor strength and if untreated, cause emotional distress, social isolation, anxiety and depression [2]. Although hormonal therapies, including systemic or local estrogen, are routine for treating GSM, their use is restricted in patients with previous hormone sensitive cancer, necessitating the development of safe, yet effective, non-hormonal therapies [3]. In novel alternatives under consideration, energy-based therapies like diode laser therapy have gained favor by encouraging vaginal tissue rejuvenation, boosting local blood supply and maintaining hydration, flexibility and function of the vagina [4]. Clinical trials have documented that laser treatment relieves symptoms of vaginal atrophy and enhances sexual function and quality of life [5]. Concurrently, pelvic floor rehabilitation, including by a pelvic floor chair, has been effective in treating urinary incontinence, a common comorbidity of GSM [6]. Combination of these modalities may provide synergistic effects, addressing sexual and urinary symptoms simultaneously. Additional evidence is, however, required to determine optimal protocols and long-term outcomes [7]. Therefore, it is of interest to compare the effectiveness of adjunctive diode vaginal laser treatment and pelvic floor chair sessions for GSM in postmenopausal women, utilising validated measuring tools to inform the evidence base for non-hormonal therapies. Through investigating this integrated model, the study aims to further develop safer, less invasive interventions that have the ability to improve the health and quality of life of women with GSM.

## Methodology:

Fifty postmenopausal women with genitourinary syndrome of menopause (GSM) ages 45-70 participated in this prospective observational study at SUN Hospital in Patna, India. Without a control group, participants received pelvic floor chair sessions in addition to diode vaginal laser therapy. Postmenopausal women within the designated age range who had been diagnosed with GSM and were not on hormone replacement treatment were eligible for inclusion. Current genital infections, recent pelvic surgery (within the last three months), hormone medication use during the previous six months, pregnancy, metallic implant presence, bleeding problems, or tumours were among the exclusion criteria. Based on previous research indicating a medium to high effect size for symptom improvement, the sample size of 50 was chosen to ensure 80% power at a 5% significance level to identify significant improvements in FSFI and ICIQ-UI SF scores. The course of treatment comprised three about 20-minute sessions of diode laser therapy spaced four weeks apart (1470 nm wavelength, 17W pulsed mode, 5 Hz, 50 ms pulse duration). A radial fibre handpiece was placed vaginally and, following the application of lidocaine gel, it delivered eight circumferential pulses per centimetre. For a total of twelve sessions, excluding laser days, pelvic floor chair sessions were held twice a week for six weeks. Each 28-minute session of the Stingray Emsella chair (up to 2.5 Tesla intensity) produced supramaximal contractions of the pelvic floor. Participants received education on pelvic floor engagement and personalised adjustments to the degree of therapy. The FSFI-19, ICIQ-UI SF and VAS scores for vaginal symptoms were used to measure the primary outcomes, which included improvements in GSM symptoms, sexual function, urine incontinence and general quality of life. Changes in the use of absorbent pads were among the secondary outcomes. At baseline and three months after therapy, all outcomes were assessed. SPSS version 23 was used to analyse the data. Clinical and demographic features were summed together using descriptive statistics. Pre- and post-treatment scores were compared using paired t-tests; p-values <0.05 were deemed statistically significant.

## Results:

Table 1 (see PDF) reports the baseline characteristics of the 50 study subjects. The participants' mean age was 58.3 years (standard deviation = 5.9). The mean BMI was 28.5 kg/m^2^ and the mean menopause duration was 8.5 years. The mean parity (number of children) was 2.1. With regard to marital status, 40 subjects (80%) were married and 10 subjects (20%) were unmarried or widowed. Just 5 patients (10%) had a background of gynecological surgery and 30 patients (60%) had a history of previous urinary infection. 12 patients (24%) had vaginal infection reported before starting the therapy, while 38 patients (76%) did not have any infection. 35 patients (70%) used non-hormonal lubricants and 15 patients (30%) did not. Figure 1 (see PDF) will depict box plots for all continuous variables while Figure 2 (see PDF) will plot the column chart to all the remaining characteristics. Table 2 (see PDF) The Visual Analogue Scale (VAS) shows improvement in the symptoms of vaginal atrophy. The mean score for vaginal dryness was 75.0 before therapy, but it dropped to 20.0 after treatment (p < 0.001). Significant improvements were also observed in vaginal burning, itching, dyspareunia and dysuria; pre-treatment ratings were 80.4, 78.2, 85.3 and 83.1, respectively, whereas post-treatment levels were 25.4, 24.2, 30.3 and 29.5 (all p < 0.001). There was an improvement in the overall VAS score from 80.4 to 25.8 (p < 0.001). Figure 3 (see PDF) is a bar chart of Vaginal Atrophy Symptoms (VAS), showing a significant improvement in VAS scores for each symptom after treatment.

Table 3 (see PDF) demonstrates the improvement in sexual function as determined by FSFI-19 scores. Significant gains were observed in every domain: overall satisfaction increased from 2.0 to 4.1, lubrication improved from 3.8 to 5.5, discomfort decreased from 2.5 to 5.0, sexual desire improved from 3.0 to 4.2, sexual arousal improved from 3.3 to 5.2 and orgasm improved from 2.8 to 4.9 (all p < 0.001). From 17.4 to 25.9, the overall FSFI score increased dramatically (p < 0.001). Sexual Function (FSFI-19) is shown in Figure 4 (see PDF), with scores rising after therapy and improvements in a number of aspects. Improvements in a number of domains are seen in Figure 4 (see PDF), along with higher post-treatment scores. Table 4 (see PDF) demonstrates the effect on the symptoms of urine incontinence as indicated by ICIQ-UI SF. Frequency improved from a pretreatment score of 4.5 to 2.1, urgency dropped from a pretreatment mean score of 5.0 to a post-treatment mean score of 2.5 and incontinence episodes likewise fell from 5.1 to 2.6, all of which were p < 0.001. Additionally, there was a significant improvement in the utilisation of absorbent pads from 3.0 to 1.1 (p < 0.001). From 14.6 to 7.2, the overall ICIQ-UI SF score dropped (p < 0.001). Urine Incontinence (ICIQ-UI SF) is shown in Figure 5 (see PDF), where a decline in urine symptom scores indicates improvement following the intervention. The baseline characteristics in Table 1 (see PDF) show a study population with an average age of 58.3 years and menopause duration of 8.5 years, with significant GSM-related symptoms, including a high prevalence of urinary infections (60%) and prior vaginal infections (24%). Table 2 (see PDF) shows a significant decrease in the symptoms of vaginal atrophy, such as dryness (VAS scores from 75.0 to 20.0) and dyspareunia (85.3 to 30.3), which show the effectiveness of the treatment (p < 0.001). Table 3 (see PDF) shows marked improvement in sexual function with the increase in FSFI scores from 17.4 to 25.9, driven by enhancements in lubrication and satisfaction (p < 0.001). Finally, Table 4 (see PDF) confirms significant alleviation of urinary incontinence symptoms, with reductions in urgency, incontinence episodes and absorbent pad usage (p < 0.001). Figure 1 (see PDF) presents a box plot that captures the distribution of continuous variables; these include age, BMI and menopausal duration with fairly homogenous characteristics across all the participants under study. A stacked column chart for categorical variables such as marital status and lubricant use is displayed in Figure 2 (see PDF), in which 80% of respondents were married and 70% were using non-hormonal lubricants. Figure 3 (see PDF): A bar chart showing significant reductions in symptoms of vaginal atrophy, with visually salient declines in dryness and dyspareunia post-treatment. Figure 4 (see PDF): Improved sexual function is represented through a bar chart, where gains are made across FSFI domains, such as lubrication and satisfaction. Finally, Figure 5 (see PDF) illustrates a decline in urinary incontinence symptoms by a bar chart, with marked declines in urgency, incontinence episodes and absorbent pad usage.

## Discussion:

This prospective observational study demonstrated significant improvements in symptoms of vaginal atrophy and overall sexual functioning among postmenopausal women with genitourinary syndrome of menopause (GSM) following treatment with diode vaginal laser therapy combined with pelvic floor chair sessions [7, 8]. Validated assessment tools, including the Female Sexual Function Index (FSFI), International Consultation on Incontinence Questionnaire Urinary Incontinence Short Form (ICIQ-UI SF) and Visual Analog Scale (VAS), ensured reliable measurement of treatment outcomes [9, 10]. Our findings align with existing literature supporting the efficacy of diode laser therapy which reported notable improvements in vaginal dryness and dyspareunia [11]. The addition of electromagnetic pelvic floor stimulation further contributed to a reduction in urinary urgency, frequency and leakage, consistent with findings on magnetic stimulation in stress urinary incontinence [12, 13]. Sexual function, particularly in domains such as lubrication, arousal and satisfaction, also showed meaningful enhancement, echoing the review on the growing role of laser therapies in vulvovaginal atrophy [14]. The FSFI score in our study rose from 17.4 ± 4.2 to 25.9 ± 5.0 post-treatment, highlighting the synergistic effect of combining therapies [15]. Similarly, study reveals the role of laser therapy in addressing urinary symptoms and its utility even in complex cases such as breast cancer survivors [16]. However, the absence of a control group limits definitive conclusions and future randomized controlled trials are needed to validate these findings. Long-term follow-up studies, are essential to assess the durability of benefits and explore potential applications in related conditions such as endometriosis or breast cancer recurrence linked to hormonal therapies.

## Conclusion:

A significant improvement in symptoms of genitourinary syndrome of menopause and sexual function among postmenopausal women with diode laser therapy combined with pelvic floor chair sessions is shown. It effectively alleviated the atrophy of vagina and urinary incontinence to enhance quality of life. However, it needs to be validated with high-quality randomized controlled trials and sustainability studies for a long duration.
